# Lifestyle of sponge symbiont phages by host prediction and correlative microscopy

**DOI:** 10.1038/s41396-021-00900-6

**Published:** 2021-02-18

**Authors:** M. T. Jahn, T. Lachnit, S. M. Markert, C. Stigloher, L. Pita, M. Ribes, B. E. Dutilh, U. Hentschel

**Affiliations:** 1 GEOMAR Helmholtz Centre for Ocean Research Kiel, Kiel, Germany; 2 Christian-Albrechts-University of Kiel, Kiel, Germany; 3 Imaging Core Facility, Biocenter, University of Würzburg, Würzburg, Germany; 4 Institut de Ciències del Mar (ICM-CSIC), Barcelona, Spain; 5 Theoretical Biology and Bioinformatics, Utrecht University, Utrecht, The Netherlands; 6 Present Address: Department of Zoology and Department of Biochemistry, University of Oxford, Oxford, UK

**Keywords:** Bacteriophages, Microbial ecology, Symbiosis

## Abstract

Bacteriophages (phages) are ubiquitous elements in nature, but their ecology and role in animals remains little understood. Sponges represent the oldest known extant animal-microbe symbiosis and are associated with dense and diverse microbial consortia. Here we investigate the tripartite interaction between phages, bacterial symbionts, and the sponge host. We combined imaging and bioinformatics to tackle important questions on who the phage hosts are and what the replication mode and spatial distribution within the animal is. This approach led to the discovery of distinct phage-microbe infection networks in sponge versus seawater microbiomes. A new correlative in situ imaging approach (‘PhageFISH-CLEM‘) localised phages within bacterial symbiont cells, but also within phagocytotically active sponge cells. We postulate that the phagocytosis of free virions by sponge cells modulates phage-bacteria ratios and ultimately controls infection dynamics. Prediction of phage replication strategies indicated a distinct pattern, where lysogeny dominates the sponge microbiome, likely fostered by sponge host-mediated virion clearance, while lysis dominates in seawater. Collectively, this work provides new insights into phage ecology within sponges, highlighting the importance of tripartite animal-phage-bacterium interplay in holobiont functioning. We anticipate that our imaging approach will be instrumental to further understanding of viral distribution and cellular association in animal hosts.

## Introduction

Marine animals are constantly exposed to viruses considering average titres of 10 million virions per millilitre of seawater [1]. Yet, viral ecology within animal-associated microbiota and its impact on animals are still poorly understood (reviewed in [2–4]). This is staggering considering the impact of viral lysis in the functioning of whole marine ecosystems, e.g. via shaping of dissolved organic matter (DOM) fluxes (viral shunt; [5]) and bacterial diversity (kill-the-winner dynamics; [6, 7]). Sequencing of viral particles from animals, including humans, provided new insights into the composition of animal-associated viral communities (i.e. viromes, [8, 9]). This revealed a trend for animal species-specific viromes [10, 11], that are individually unique and stable over time [12–14].

Bacteriophages (phages) dominate seawater viral communities, and are estimated to lyse 20–50% of marine surface bacteria per day [15]. However, while lytic bacteriophages act as important bacterial killers in the oceans, temperate phages may integrate into bacterial genomes linking their fates as a lysogen. Indeed, about half of the marine bacteria encode phages in their genomic repertoire [16, 17]. Importantly, the integrated phage then benefits from enhancing the fitness of its bacterial host as now they multiply together [18, 19]. Therefore, lysogenic conversion mechanisms provide a selective advantage to phages, significantly extending their role as bacterial killers to providers of novel beneficial functions (reviewed in Howard-Varona et al. [18]). This is confirmed by exciting examples showing that prophages can indeed enhance the protection of their bacterial hosts such as by providing superinfection exclusion [20] or by providing virulence factors to pathogens (e.g. Shiga toxins; [21]) extending their bacterial host niches.

Research on phage replication strategies in animal hosts is so far primarily focussed on the guts of humans (20–50% temperate; [3]), mice (temperate dominance; [22, 23]), and honey bees (lytic dominance; Bonilla-Rosso et al. [24]). In animal-associated microbiomes, phages and eukaryote host cells may interact either directly or indirectly. Direct interactions include e.g. phagocytosis of phage virions by eukaryote cells, as reviewed in Van Belleghem et al. [4], while indirect interaction occurs via manipulation of the microbiome by the phage or eukaryote. In this context, we recently discovered a novel symbiont phage-encoded protein in sponges (ANKp) that modulates eukaryote–bacterium interaction by altering the eukaryotes’ response to bacteria and which seems to be widespread in eukaryote host associated systems [12]. Further, *Pseudomonas aeruginosa* phage RNA was reported to subvert the eukaryotic immune response leading to reduced pathogen clearance [25]. Together, these insights formulate an emerging paradigm that certain phages can modulate the eukaryote immune system via tailored effectors. Knowing the replication strategies of phages inside animals is, therefore, an important proxy for their effects on the ecology and evolution of host-associated microbes.

The present study aims to shed light on the lifestyle of phages within marine sponge holobionts. Marine sponges are sessile filter feeders that are massively exposed to planktonic microbes, including viruses, translating to roughly 56 billion virions filtered by a sponge per day [26, 27]. Despite such high exposure rates to seawater microorganisms, they contain highly host species-specific microbial [28, 29] and viral [12, 30] communities. Sponge microbiology spans all domains of life including Bacteria, Eukaryotes (e.g. unicellular algae, protists and fungi) and Archaea [31]. Sponge viromes are dominated by clades of bacteriophages, such as tailed bacteriophages of the order *Caudovirales* and tailless *Microviridae* [12, 30, 32]. However, since viromes are generated by physical separation of viruses prior to sequencing, they lack a potentially critical link to their spatial niche and the bacteria that they infect. In order to resolve phage ecology in the sponge holobiont, we deemed it necessary to develop a new microscopy approach that would complement bioinformatic predictions. Available visualisation methods were found to be insufficient as electron microscopy does not easily allow the sequence-based identification of specific viruses and fluorescence in situ hybridisation of phages [33] lacks the subcellular context needed to visualise phages in the animal tissue. In the present study, we report on (i) phage-microbe infection networks in sponges and seawater, (ii) a new correlative phageFISH approach allowing the visualisation of phages in the native animal context, and (iii) phage replication strategies within sponges. Collectively, our work uncovers phages as central elements of the microbial ecology within marine sponges.

## Methods

### Integrative host prediction

We predicted the bacterial hosts of marine sponge-derived phages through a combination of prognostic computational approaches via CRISPR-spacer-, tRNA and genome homology, as reviewed in Edwards et al. [34]. We screened 4484 sponge associated viral sequences (≥5 kb, hereafter ‘BCvir’) previously published by Jahn et al. [12] against a custom database representing sponge-associated microbial symbionts and planktonic microbes (X2, hereafter). This database is comprised of microbial sequences from three Mediterranean sponges (*Petrosia ficiformis*, *Sarcotragus foetidus*, *Aplysina aerophoba*; [35]), 37 high quality bins from *A. aerophoba* [36], and assembled microbial metatranscriptomes (*Xestospongia muta* [37]; *Xestospongia testudinaria*; [38]). This set was augmented with 290 Tara Oceans metagenome-assembled genomes from the Mediterranean Sea [39] and all 97,941 bacterial genomes available in PATRIC (as of June 2017). **CRISPR-match**. The X2 database was searched for CRISPR spacers using CRISPRDetect_2.2 (-array_quality_score_cutoff 3 -q 1, Biswas et al. [40]). The identified spacers were subsequently matched to BCvir contigs by BLASTn search (-dust no -gapopen 10 -gapextend 10 -penalty -1 -e-value 1 -word_size 7) as suggested by Biswas et al. [41]. Hits were allowed a maximum of 1 mismatch over full spacer length to increase stringency against false positive classification [42]. **Homology match**. Viral BCvir genomic signatures in microbial genomes, i.e. lysogens, were identified via a search against the X2 database through BLASTn. The best hits below an e-value threshold of 10^−5^ were considered a match when phages aligned with more than 80% sequence identity over a length between 1 kb and 50% of the microbial host contig. **tRNA match**. tRNA sequences predicted in BCvir contigs using tRNAscan-SE v.1.23 [43] with default settings and searched against X2 using BLASTn keeping only best hits with at least 95% sequence identity. All predictions were combined in an ensemble infection network that was visualised via Cytoscape v3.6.0 [44].

### Phage replication mode

The lytic or lysogenic lifestyle of sponge associated phages was predicted using a combination of the pre-trained supervised random forest classifier implemented in the Phage Classification Tool Set (PHACTS; [45]), and hits to prophage marker enzymes (i.e., integrase or excisionase; PF00589, PF02899, PF13102, PF13356) searching the pVOG database [46] with HMMER 3.1b2 (hmmscan -E 10-5) or InterProScan v5.27-66.0 [47].

### Sponge sampling and processing

The sponge *Aplysina aerophoba* was collected for imaging at Cala d’Aiguafreda, (3°13′39.8′′E, 41°57′51.3′′N; October 2016), Begur, Spain by snorkelling. We randomly sampled three phenotypically healthy individuals in a radius of 20 m (see Supplementary Table S1). Sponge tissue dissection, high-pressure freezing and freeze-substitution were carried out as described in Jahn et al. [48]. Briefly, we dissected standardised (2 mm × 200 µm) pinacoderm (=outer layer, *n* = 3 per individual) and mesohyl (=inner tissue, *n* = 3 per individuum) samples. Samples were then immediately cryo-immobilised at >20,000 K/s freezing speed and >2100 bar pressure via high pressure freezing (EM HPM100, Leica Microsystems GmbH, Wetzlar, Germany). Following freeze-substitution (AFS2; Leica Microsystems, Wetzlar, Germany), samples were embedded in LR-White (London Resin Company Ltd.)

### Phage fluorescence in situ hybridisation-correlative light and electron microscopy (PhageFISH-CLEM)

We modified and extended the ViewHIV approach of Chin et al. [49] which detects the human immunodeficiency virus in laboratory cell cultures. The modification was undertaken to localise virions and integrated prophages of the predicted bacteriophages in their natural context within cryo-immobilised and freeze-substituted sponge tissues. The extension by correlative light and electron microscopy (CLEM) allowed the localisation of the phage fluorescence spots within their native structural context. Detailed protocols for PhageFISH-CLEM staining are made available at the open-access repository of science methods protocols.io (10.17504/protocols.io.krdcv26). The staining approach was established and validated on the test-system of (1) *Curvibacter* sp. AEP, encoding a prophage (2) *Curvibacter* sp. Hvul, not encoding this prophage, and (3) the purified phage virion (Supplementary Fig. S1). Staining with the probes was performed according to the manufacturer’s instructions (VIEWRNA CELL PLUS ASSAY kit; Invitrogen, Cat nr. 88-19000-99) with some modifications. Briefly, reactions were performed on 100 nm LR-White sections of embedded sponge tissue that were cut as serial ribbons using a Histo Jumbo Diamond Knife (Diatome AG, Biel, Switzerland) and deposited on poly-L-lysine coated glass slides (Polysine slides, Thermo Fisher Scientific, Waltham, MA, USA). Sections were then encircled with a hydrophobic barrier pen (Liquid Blocker, Japan) and air dried for at least 1 h. To extend the protocol for dsDNA phage targets, we added an initial denaturation step by incubating the sample with 75% formamide (Sigma) in 2× SSC for 10 min at 70 °C. The samples were then dehydrated in an ethanol series of 1 × 70%, 1 × 85% and 2 × 100% for 2 min each. Hybridisation, pre-amplification, amplification and labelling were performed according to the manufacturer’s instructions in a humidified chamber placed in a hybridisation oven (OV5, Biometra, Göttingen, Germany). The non-target AEP probe and buffer controls were added as negative controls for each sponge sample. Additionally, the AEP probe was hybridised to an AEP pellet to control for the appropriate experimental conditions to allow sensitive viral detection.

### Phage probe design

For each phage of interest, an RNAview probe set consisting of 30–54 target specific oligonucleotides (Type 1: Alexa Fluor 546) was obtained from Invitrogen (Supplementary Data 6). Descriptive regions on the phage genomes that would allow discrimination against the background of other viruses and microbes of the communities were identified using permissive BLASTn v 2.2.28+ (-e-value 100) against the X2 database. By excluding matched “un-specificity” sites, we used GenePROBER (http://kronos.icbm.uni-oldenburg.de/shiny/web-probe-designer/, options GC 40-70; A 25; B 70; C 95; D 95; E 95; G 93, H 93; I 2; J 2; K 5; L 5; M 40; N 1; O 0.05) to design probes against structural phage genes with low mutation rates. The established probe sets (see Supplementary Table S2 for all sequences) are available upon request.

### Correlative light and electron microscopy and alignment

PhageFISH signals were detected using an Axio Observer.Z1 microscope, equipped with AxioCam 506 and Zen 2 version 2.0.0.0 (Carl Zeiss Microscopy GmbH, Göttingen, Germany). Sponge tissue regions of interest were catalogued with a zoom-out reference catalogue to facilitate re-observing the same regions at the electron microscope as follows: acquisition of region of interest (ROI) using ×63 objective, acquisition of ROI using 40x objective, acquisition of ROI using ×20 objective, acquisition of ROI using ×10 objective, stitching of whole section using ×10 objective. Slides were then processed as detailed in Jahn et al. [48]. Briefly, cover glasses were lifted without lateral movement using a razor blade, and Mowiol mountant (Mowiol 40–88, Kuraray Europe GmbH, Tokyo, Japan) was washed off for 2 × 5 min with PBS. The sections were dried and contrasted in 2.5% uranyl acetate in ethanol for 15 min and in 50% Reynolds’ lead citrate [50] in decocted ddH_2_O for 10 min. The slides were size-reduced to the region of the sections using a diamond pen and attached to a scanning electron microscopy (SEM) pin stub specimen mount. After coating the sample with a ca. 2.5 nm thick carbon layer to prevent charging of the sample (CCU-010 coating unit with CT-010 carbon thread head, Safematic, Switzerland), the samples were ready for imaging using a field emission scanning electron microscope JSM-7500F (JEOL, Japan) with LABE detector (imaging of low angle backscattered electrons). Using the zoom-out reference catalogue described above, regions of fluorescence microscopy were identified at SEM resolution and were correlated using the eC-CLEM tool [51] based on DAPI heterochromatin patterns that are visible in both imaging modalities. Probe signals were quantified with optimised signal-to-noise threshold (Supplementary Fig. S2) using the MaximumFinder function in ImageJ v.1.52 [52]. We note that these estimates are not absolute but rather relative measures of abundance as stated in Chin et al. [49].

### Statistical analyses

We compared the topology of the complete infection network against 10,000 networks containing randomised labels. Therefore, the test statistic was obtained from the network using mosaicCore package v0.6.0 and was compared against the distribution of randomisation statistics (Supplementary Fig. S3). Statistical significance between tissues and targeted phages in imaging data and between phage replication strategies was determined using Kruskal–Wallis tests followed by Dunn’s Post-hoc-Tests with Benjamini–Hochberg false discovery rate correction. Throughout, *p* values < 0.05 were considered statistically significant. Statistical tests were performed using R version 4.0.0 [53]. Statistical outputs are summarised in Supplementary Tables S3, S4.

## Results and discussion

### Distinct phage-bacteria infection networks within marine sponges

**Fig. 1:**
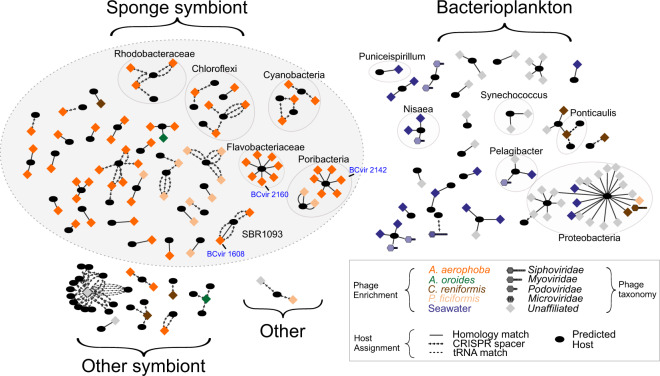
Predicted phage–bacteria infection networks. Phage (diamond) assignments to putative bacterial hosts (oval) by combining signals from CRISPR-spacer-, homology- and tRNA matches against a custom database of sponge microbial sequences, PATRIC genomes [81] and 290 Tara Oceans bins from bacterioplankton of the Mediterranean Sea (TMED; Tully et al. [39]). Nodes are organised in groups by niche of the predicted host. ‘Other symbionts’ were isolated from eukaryote hosts including fish and sea cucumber. Phage taxonomy was assigned using reticulate classification based on gene sharing with ViralRefseq entries as performed in Jahn et al. [12]. The symbiont phages subjected to imaging in the next section are labelled in blue text. BCvir 2312 and adjacent nodes were excluded from this representation to aid visualisation. A full network is provided in Supplementary Fig. S4; the underlying raw data are provided in Supplementary Table S5.

Using integrative in silico prediction, we established the first connections between sponge-associated phages and the bacteria they infect. Overall, this approach matched 142 BCvir (3.2%; 142 of 4484) phage genomic sequences to 154 bacterial genomes and 29 metagenomic contigs (full network, Supplementary Table S5). The predicted phage hosts (Fig. 1) comprised representatives from cosmopolitan sponge symbionts such as *Poribacteria*, *Cyanobacteria*, *Chloroflexi* and *Flavobacteria* [54, 55]. On the other hand, predicted bacterioplankton hosts included Candidatus *Puniceispirillum* (TMED52; Tully et al. [39]) and *Synechococcus* sp. (TMED20). The specificity of our predictions was confirmed by scarce matching to the added genomic background of ~ 97,000 genomes representing expected non-target hosts from other environments (0.03%; 34 of 97,941).

Notably, the majority of phages predicted to infect sponge symbionts (74 of 76; symbiont phages, hereafter) were also enriched in sponges (Table 1). In contrast, all seawater-enriched phages were also assigned to planktonic bacteria whilst being depleted in sponges (24 of 24; planktophages, hereafter). This stratification of the infection network to either sponge or seawater was significant when compared to null models of independent random assortment (*p* value ≤ 0.0021; test statistic ≤ −0.1934260 for 10,000 randomisations, see Supplementary Fig. S3). This differentiation was also reflected on the level of taxonomic novelty. Viral clusters (VCs) are groups of viral genomes that share more genes than can be expected by chance [56]. While most predicted sponge symbiont phages belonged to novel VCs [12] that are exclusively found in sponges (76.1%; 51 of 67; Supplementary Fig. S4a), most planktophages constituted VCs already described from seawater (96,8%; 61 of 63). Together, this indicates that the sponge microbiome provides a unique niche for novel phages based on their specificity to sponge symbionts. This observation is consistent with our previous finding that the composition of sponge viromes largely mirrors that of the corresponding sponge-associated microbial consortia [12].

**Table 1:** Environmental partitioning of the phage-host infection network.

	Bacterial source			
Phage source	Sponge	Seawater	Other environment	Other animal
Sponge	74	8	2	10
Plankton	0	24	0	0
Mixed	2	67	33	38

Yet, some exceptions from this theme were noted for planktophages. First, phages with predicted planktonic microbial hosts (e.g. *Proteobacteria*) were enriched in the sponges *Chondrosia* and *Petrosia* (Fig. 1, right). Second, the majority of planktophages was present in both seawater and sponges (67.7%; 67 of 99; Table 1, mixed)*. Chondrosia* and *Petrosia* both share the same environment and a compact body plan with a finely branched aquiferous system and small choanocyte chambers [57]. It is conceivable that the different sponge morphologies [58] could impact on the sponges’ ability to retain planktophages; while beyond the scope of the present study, this possibility warrants further investigation. An alternative explanation could be that bacterial lysis by phage is induced upon stress encountered when entering the sponge. Switching of phage replication from lysogenic to lytic mode is well known to be induced by stress, such as mediated by chemicals and nutrients [59], leading to the activation of DNA damage response (SOS response; [60]. The predicted lysogenic planktophage BCvir 3312 (Supplementary Fig. S4b), that occurs in sponges would represent a case fitting to such a scenario. In analogy to the ‘Bacteriophage Adherence to Mucus’ model (BAM; Barr et al. [61]), this might suggest that phages use sponges as hunting grounds to infect planktonic microbes that are concentrated within sponges via filtration activity of the animal. Our phage host prediction rate of 3.2% (142 of 4484) is similar to the 5% stated by Roux [62] which is based on the largest viral genome repository (IMG/VR v.2.0; [63]). Therefore, targeted high throughput screening strategies, such as viral tagging [64, 65] or proximity ligation assays [66] will be instrumental to validate and complement our in silico predictions of phage-microbe pairings in sponges.

The topology of our infection network highlights some phages that infect multiple bacterial hosts and, similarly, some bacterial host species that are infected by multiple phages (Fig. 1, Supplementary Fig. S5). Generally, phage infectivity is considered to be limited to particular bacterial species or even particular strains [67]. However, recent evidence indicates that broad-host-range phages (i.e. polyvalent phages) are more widespread than previously thought [68, 69]. Whilst such conclusions in marine ecosystems are mainly drawn from planktonic environments [69, 70] and corals [71], we add here in silico evidence suggesting that broad-host range phages may also be present in sponges, and possibly other filter feeding invertebrates. These broad-host range phages are known to foster horizontal gene transfer [72] and likely play a role in the evolution of sponge associated microbes.

### A new imaging approach, ‘PhageFISH-CLEM’ reveals clearance of phages by sponge cells

**Fig. 2:**
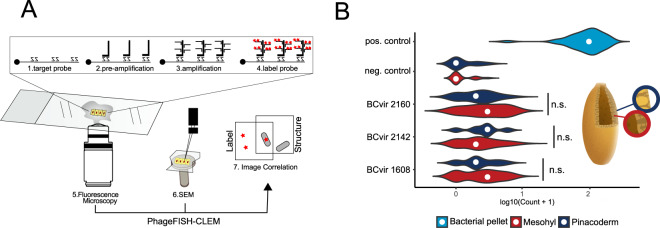
PhageFISH-CLEM allows automated tissue-wide quantification of phage signals. **A** Scheme illustrating Phage-FISH-CLEM. **B** Computer-aided image quantification of phage signals reveals abundances of different phages over tissues (pinacoderm vs. mesohyl). The graph represents values for *n* = 41 (pos.control, lysogen pellet), 43 (neg.control, non-lysogen pellet), 36 (BCvir 2160; Flavobacteria phage), 66 (BCvir 2142; Poribacteria phage), 98 (BCvir 1608; SBR1093;EC214 phage) measurements of two individuals. Credit: sponge illustration Kelvinsong/Wikimedia Commons. Related Supplementary Figs. S1, S2 for method validation and Supplementary Table S3, S4 for statistics.

**Fig. 3:**
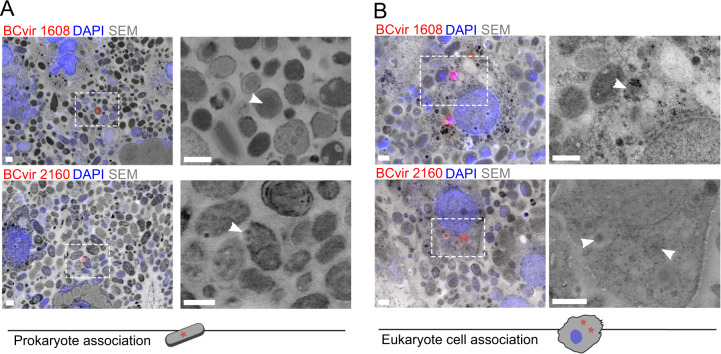
Correlative light and electron microscopy localises phages to host ultrastructure. Representative micrographs localise symbiont phages to **A** their bacterial symbiont hosts and **B** the sponge phagosomes. White boxes indicate the regions that are magnified on the right and arrows denote positions of phage signal. Scale bars 1 µm. For correlation statistics and single channels see Supplementary Fig. S6.

Surprisingly, no approach exists to our knowledge to visualise specific viruses in animal tissues at subcellular resolution by closing the gap between phageFISH and electron microscopy. We therefore established a new microscopy protocol, termed PhageFISH-CLEM (Fig. 2A; for details see Methods), that allows not only the quantification (Fig. 2B), but also the visualisation of virus-cell associations (Fig. 3) at electron microscopy resolution within tissues. Initially, the previously established branched DNA amplification approach [73] was tested in pilot-experiments using the well characterised *Hydra* symbiont lysogen/non-lysogen system *Curvibacter* sp. AEP/Hvul [74]. This protocol allowed us to specifically localise both virions and integrated prophages at single molecule sensitivity (Supplementary Figs. S1, S2). Notably, this approach covers the whole spectrum single- and double- stranded DNA and RNA viruses in host tissues, thus being widely applicable in the field of environmental virology.

To further elucidate the phage lifestyle within the sponge landscape we applied PhageFISH-CLEM on cryo-immobilised and freeze substituted *A. aerophoba* sponge tissues. Three phages were selected (BCvir 2142, BCvir 1608, BCvir 2160) that were both abundant and predicted to infect cosmopolitan sponge symbionts Poribacteria, SBR1093; EC214 and Flavobacteria, respectively (Fig. 1, blue, Supplementary Table S2). Probe signals of all three phages could be identified in the mesohyl interior as well as in the outer pinacoderm (Fig. 2B, non-target probe comparison; *p* values ≤ 0.0007, *z* ≥ 3.32, df = 4; details in Supplementary Table S3). Notably, samples for imaging were obtained from different timepoints and locations than the samples for sequencing (see Supplementary Table S1) highlighting that the assayed symbiont phages can be stably detected 84 days after virome sampling. Further, differences in phage signals between BCvir 2142, BCvir 1608 and BCvir 2160 were marginal (KW test: kw chi squared = 101.5033, df = 4; *p* value ≥ 0.4437) indicating similar abundances of the assayed symbiont phages in the sponge tissue. Computer-aided quantification (*n* = 200 images; screened area 39,788 × 31,922 µm) of phage signals revealed no clear delineation of phage signal levels between the outer pinacoderm and inner mesohyl tissue in general (Kruskal–Wallis (KW) test: *p* value = 0.6312, chi squared = 0.23044, df = 1), and when tested for the three phages separately (Supplementary Table S4). The detected similar abundances of phages between the outer pinacoderm layer and the inner sponge mesohyl is consistent with our previous findings that were obtained by viral community sequencing [12].

We next analysed the association of phages with cells within the inner mesohyl tissue by high resolution correlated light and electron microscopy (CLEM). This revealed two main phage loci (Fig. 3A, B, Supplementary Fig. S6): First, signals for each of the three assayed phages appeared as single spots located predominantly within bacterial cells (Fig. 3A), which is indicative of their lysogenic or pseudo-lysogenic stages. This lifestyle is further supported by the presence of integrase genes in BCvir 2160, and by homologies to integrated prophages of symbiont genomes for all three phages (Fig. 1). Furthermore, morphotypes of targeted prokaryote cells differed per phage probe, which is consistent with the different bacterial hosts they were predicted for. Second, BCvir 1608 and BCvir 2160 signals gravitated towards sponge cells, while this was not detectable for BCvir 2141. Correlated electron microscopy of these regions confirmed their intracellular localisation within phagosomes of sponge cells (Fig. 3B), which are vesicles containing engulfed particles. The size of measurable particles within the phagosomes ranged around 200 nm (average 197.7 nm, ±SD 20.4, *n* = 5), indicating that virions rather than lysogens were cleared by sponge phagocytosis. This is a notable observation which coincides with the generally low phage particle abundances in the extracellular host matrix reported here (Fig. 3B, C) and elsewhere [75]. Therefore, we postulate that the sponge cell phagocytosis reduces the amount of free phage virions in the sponge matrix, which may critically modulate the infection dynamics between phages and their bacterial symbiont hosts.

### Symbiont phage lysogeny dominates the sponge matrix

**Fig. 4:**
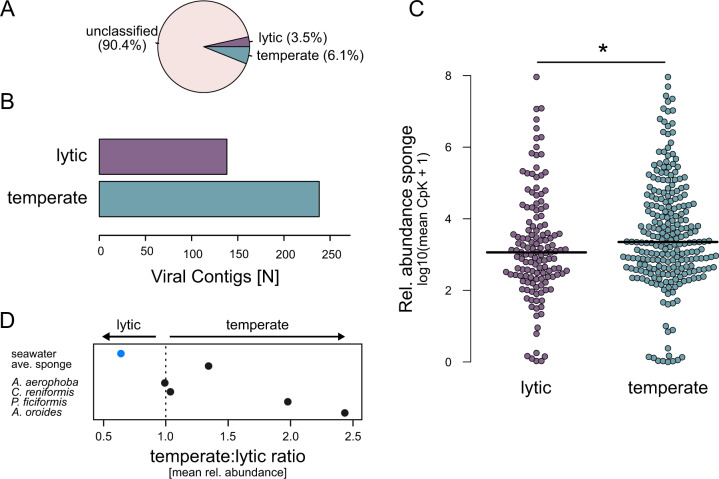
Lifestyle prediction of sponge enriched phages. **A** Classifications for *N* = 3936 sponge associated viruses. **B** Total number of phages predicted for each category. **C** Rel. abundance of predicted phage lifestyle categories in sponges; Cpk, Copy per kilobase. **D** Relative abundance ratios of lytic versus lysogenic phages by environment.

We hypothesised that host mediated virion clearance would favour lysogenic replication as discussed for phages in harsh extracellular conditions [19, 76]. To test this hypothesis, we screened BCvir phages for temperate phage markers (i.e. integrases, excisionases) and supplemented this approach with Random-Forrest based predictions (Fig. 4A, Methods). Interestingly, based on a fraction of 9.6% of BCvir phages with replication mode predictions (Fig. 4A), lysogenic replication seems indeed to be the more prevalent replication mode among sponge symbiont phages. This is indicated by a 1.7 (239/138) times higher prevalence of temperate phage contigs (Fig. 4B), along with a significantly higher relative abundance (KW test: *p* value = 0.01007, chi squared = 6.6223, df = 1) as compared to predicted lytic phages (Fig. 4C).

To apply a more comprehensive analysis, we then compared relative abundances of temperate to lytic ratios (T:L ratio) in seawater phages and symbiont phages (Fig. 4D). Lytic strategies dominated in seawater phages, whereas the *P. ficiformis* and *A. oroides* associated phages were enriched for lysogenic phages, while in *A. aerophoba* and *C. reniformis* lytic and temperate phages appeared in equal proportions. When we cleaned the sponge data from planktophages, a temperate lifestyle became apparent for phages in all assayed sponge species. We propose three factors that may favour lysogenic strategies in sponges. First, lysogeny is favoured under conditions when virion decay rates are high [19], which agrees with the pronounced virion phagocytosis by sponge archaeocytes (PhageFISH-CLEM, previous paragraph). Second, the prevalence of lysogens would be consistent with the Piggyback-the-Winner model for systems with high microbial densities [77], as it is the case for our tested sponge species. Ultimately, widespread lysogeny offers a high genomic potential to foster bacterial symbiont adaptation via horizontal transfer of auxiliary genes reported earlier [12].

Notably, the phage sequences used in this study are derived from purified virus-like particles. Therefore, since great care was taken to minimise sampling derived stress induction by instant freezing in the field (Methods), we are confident that lytic events triggered by temperate phages are widespread in all sponge individuals under native conditions. Lysis of specific bacterial symbionts can favour surviving bacterial competitors [18, 74, 78]. Via kill-the-winner dynamics [6, 79] this might facilitate the excessive diversity known from sponge microbiomes [55, 80]. Similar mechanisms might impact the physiology within holobionts representing exciting routes for further research.

## Conclusions

**Fig. 5:**
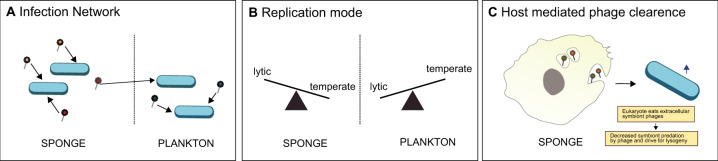
Conceptual diagram summarising insights into the ecology of sponge-associated phages. **A** Different phage-bacteria infection networks between sponges and seawater. **B** Temperate phages are more prevalent in sponges than in nearby seawater. **C** Potential impact of phage phagocytosis by sponge cells on phage-symbiont interactions.

We resolve the ecology of phages in the context of the sponge host and its bacterial symbionts. We report on two main findings: (i) Sponges maintain surprisingly confined and specific phage-bacteria infection networks, despite the constant exposure to planktophages. While our predictions, including the discovery of cosmopolitan sponge symbionts, are likely to underestimate the true complexity of these phage-host pairings, this data provides an important baseline for future studies on the regulatory role of phages in the holobiont. (ii) Based on our new PhageFISH-CLEM imaging approach, we observed virion phagocytosis by sponge cells, indicating that the sponge host modulates phage-bacteria ratios. This behaviour ultimately favours lysogenic phage replication in sponges (Fig. 5). Collectively, our findings unravel an adapted phage lifestyle in sponges and underline the importance of tripartite animal–bacteria–phage interplay in holobiont function.

## Supplementary information

Supplementary Information

Table S2

Table S5

## References

[1] Wommack KE, Colwell RR (2000). Virioplankton: viruses in aquatic ecosystems. Microbiol Mol Biol Rev.

[2] Keen EC, Dantas G (2018). Close encounters of three kinds: bacteriophages, commensal bacteria, and host immunity. Trends Microbiol.

[3] Sausset R, Petit MA, Gaboriau-Routhiau V, De Paepe M (2020). New insights into intestinal phages. Mucosal Immunol.

[4] Van Belleghem J, Dąbrowska K, Vaneechoutte M, Barr J, Bollyky P (2018). Interactions between bacteriophage, bacteria, and the mammalian immune system. Viruses.

[5] Wilhelm SW, Suttle CA (1999). Viruses and nutrient cycles in the sea: viruses play critical roles in the structure and function of aquatic food webs. BioScience.

[6] Thingstad TF (2000). Elements of a theory for the mechanisms controlling abundance, diversity, and biogeochemical role of lytic bacterial viruses in aquatic systems. Limnol Oceanogr.

[7] Winter C, Bouvier T, Weinbauer MG, Thingstad TF (2010). Trade-offs between competition and defense specialists among unicellular planktonic organisms: the “killing the winner” hypothesis revisited. Microbiol Mol Biol Rev.

[8] Minot S, Bryson A, Chehoud C, Wu GD, Lewis JD, Bushman FD (2013). Rapid evolution of the human gut virome. PNAS.

[9] Thurber RV, Haynes M, Breitbart M, Wegley L, Rohwer F. Laboratory procedures to generate viral metagenomes. Nat Protocols. 2009;4:470–83.. 10.1038/nprot.2009.10 19300441

[10] Leigh BA, Bordenstein SR, Brooks AW, Mikaelyan A, Bordenstein SR (2018). Finer-scale phylosymbiosis: insights from insect viromes. mSystems.

[11] Wille M, Shi M, Klaassen M, Hurt AC, Holmes EC (2019). Virome heterogeneity and connectivity in waterfowl and shorebird communities. ISME J.

[12] Jahn MT, Arkhipova K, Markert SM, Stigloher C, Lachnit T, Pita L (2019). A phage protein aids bacterial symbionts in eukaryote immune evasion. Cell Host Microbe..

[13] Leigh BA, Djurhuus A, Breitbart M, Dishaw LJ (2018). The gut virome of the protochordate model organism, *Ciona intestinalis* subtype A. Virus Res.

[14] Shkoporov AN, Clooney AG, Sutton TDS, Ryan FJ, Daly KM, Nolan JA (2019). The human gut virome is highly diverse, stable, and individual specific. Cell Host Microbe.

[15] Fuhrman JA (1999). Marine viruses and their biogeochemical and ecological effects. Nature.

[16] Paul JH (2008). Prophages in marine bacteria: dangerous molecular time bombs or the key to survival in the seas?. ISME J.

[17] Touchon M, Bernheim A, Rocha EP (2016). Genetic and life-history traits associated with the distribution of prophages in bacteria. ISME J.

[18] Howard-Varona C, Hargreaves KR, Abedon ST, Sullivan MB (2017). Lysogeny in nature: mechanisms, impact and ecology of temperate phages. ISME J.

[19] Weitz JS. Quantitative viral ecology dynamics of viruses and their microbial hosts. Princeton: Princeton University Press; 2015.

[20] Bondy-Denomy J, Qian J, Westra ER, Buckling A, Guttman DS, Davidson AR (2016). Prophages mediate defense against phage infection through diverse mechanisms. ISME J.

[21] Herold S, Karch H, Schmidt H (2004). Shiga toxin-encoding bacteriophages–genomes in motion. Int J Med Microbiol.

[22] Kim M-S, Bae J-W (2018). Lysogeny is prevalent and widely distributed in the murine gut microbiota. ISME J.

[23] Reyes A, Wu M, McNulty NP, Rohwer FL, Gordon JI. Gnotobiotic mouse model of phage–bacterial host dynamics in the human gut. PNAS. 2013;110:20236–41. 10.1073/pnas.1319470110 PMC3864308 24259713

[24] Bonilla-Rosso G, Steiner T, Wichmann F, Bexkens E, Engel P (2020). Honey bees harbor a diverse gut virome engaging in nested strain-level interactions with the microbiota. PNAS.

[25] Sweere JM, Van Belleghem JD, Ishak H, Bach MS, Popescu M, Sunkari V (2019). Bacteriophage trigger antiviral immunity and prevent clearance of bacterial infection. Science.

[26] Hadas E, Marie D, Shpigel M, Ilan M (2006). Virus predation by sponges is a new nutrient-flow pathway in coral reef food webs. Limnol Oceanogr.

[27] Rix L, Ribes M, Coma R, Jahn MT, de Goeij JM, van Oevelen D (2020). Heterotrophy in the earliest gut: a single-cell view of heterotrophic carbon and nitrogen assimilation in sponge-microbe symbioses. ISME J..

[28] Lurgi M, Thomas T, Wemheuer B, Webster NS, Montoya JM. Modularity and predicted functions of the global sponge-microbiome network. Nat Commun. 2019; 10. 10.1038/s41467-019-08925-4. 10.1038/s41467-019-08925-4 PMC6397258 30824706

[29] Reveillaud J, Maignien L, Eren AM, Huber JA, Apprill A, Sogin ML (2014). Host-specificity among abundant and rare taxa in the sponge microbiome. ISME J.

[30] Laffy PW, Wood-Charlson EM, Turaev D, Jutz S, Pascelli C, Botte ES (2018). Reef invertebrate viromics: diversity, host specificity and functional capacity.. Environ Microbiol..

[31] Taylor MW, Radax R, Steger D, Wagner M (2007). Sponge-associated microorganisms: evolution, ecology, and biotechnological potential. Microbiol Mol Biol Rev.

[32] Pascelli C, Laffy PW, Botté E, Kupresanin M, Rattei T, Lurgi M (2020). Viral ecogenomics across the Porifera. Microbiome.

[33] Allers E, Moraru C, Duhaime MB, Beneze E, Solonenko N, Barrero-Canosa J (2013). Single-cell and population level viral infection dynamics revealed by phageFISH, a method to visualize intracellular and free viruses. Environ Microbiol.

[34] Edwards RA, McNair K, Faust K, Raes J, Dutilh BE (2016). Computational approaches to predict bacteriophage-host relationships. FEMS Microbiol Rev.

[35] Horn H, Slaby BM, Jahn MT, Bayer K, Moitinho-Silva L, Forster F (2016). An enrichment of CRISPR and other defense-related features in marine sponge-associated microbial metagenomes. Front Microbiol.

[36] Slaby BM, Hackl T, Horn H, Bayer K, Hentschel U (2017). Metagenomic binning of a marine sponge microbiome reveals unity in defense but metabolic specialization. ISME J.

[37] Fiore CL, Labrie M, Jarett JK, Lesser MP. Transcriptional activity of the giant barrel sponge, *Xestospongia muta* Holobiont: molecular evidence for metabolic interchange. Front Microbiol. 2015; 6. 10.3389/fmicb.2015.00364. 10.3389/fmicb.2015.00364 PMC4412061 25972851

[38] Ryu T, Seridi L, Moitinho-Silva L, Oates M, Liew YJ, Mavromatis C (2016). Hologenome analysis of two marine sponges with different microbiomes. BMC Genom.

[39] Tully BJ, Sachdeva R, Graham ED, Heidelberg JF (2017). 290 metagenome-assembled genomes from the Mediterranean Sea: a resource for marine microbiology. PeerJ.

[40] Biswas A, Staals RHJ, Morales SE, Fineran PC, Brown CM (2016). CRISPRDetect: a flexible algorithm to define CRISPR arrays. BMC Genom.

[41] Biswas A, Gagnon JN, Brouns SJ, Fineran PC, Brown CM (2013). CRISPRTarget: bioinformatic prediction and analysis of crRNA targets. RNA Biol.

[42] Burstein D, Harrington LB, Strutt SC, Probst AJ, Anantharaman K, Thomas BC (2016). New CRISPR–Cas systems from uncultivated microbes. Nature.

[43] Lowe TM, Chan PP (2016). tRNAscan-SE On-line: integrating search and context for analysis of transfer RNA genes. Nucleic Acids Res.

[44] Shannon P, Markiel A, Ozier O, Baliga NS, Wang JT, Ramage D (2003). Cytoscape: a software environment for integrated models of biomolecular interaction networks. Genome Res.

[45] McNair K, Bailey BA, Edwards RA (2012). PHACTS, a computational approach to classifying the lifestyle of phages. Bioinformatics.

[46] Grazziotin AL, Koonin EV, Kristensen DM (2017). Prokaryotic Virus Orthologous Groups (pVOGs): a resource for comparative genomics and protein family annotation. Nucleic Acids Res.

[47] Jones P, Binns D, Chang HY, Fraser M, Li W, McAnulla C (2014). InterProScan 5: genome-scale protein function classification. Bioinformatics..

[48] Jahn MT, Markert SM, Ryu T, Ravasi T, Stigloher C, Hentschel U (2016). Shedding light on cell compartmentation in the candidate phylum Poribacteria by high resolution visualisation and transcriptional profiling. Sci Rep.

[49] Chin CR, Perreira JM, Savidis G, Portmann JM, Aker AM, Feeley EM (2015). Direct visualization of HIV-1 replication intermediates shows that capsid and CPSF6 modulate HIV-1 intra-nuclear invasion and integration. Cell Rep.

[50] Reynolds ES (1963). The use of lead citrate at high pH as an electron-opaque stain in electron microscopy. J Cell Biol.

[51] Paul-Gilloteaux P, Heiligenstein X, Belle M, Domart MC, Larijani B, Collinson L (2017). eC-CLEM: flexible multidimensional registration software for correlative microscopies. Nat Methods.

[52] Schneider CA, Rasband WS, Eliceiri KW (2012). NIH Image to ImageJ: 25 years of image analysis. Nat Methods.

[53] R Development Core Team. R: a language and environment for statistical computing. In: Computing RFfS (ed): Vienna, Austria 2020.

[54] Bayer K, Jahn MT, Slaby BM, Moitinho-Silva L, Hentschel U (2018). Marine sponges as *Chloroflexi h*ot spots: genomic insights and high-resolution visualization of an abundant and diverse symbiotic clade. mSystems.

[55] Thomas T, Moitinho-Silva L, Lurgi M, Bjork JR, Easson C, Astudillo-Garcia C (2016). Diversity, structure and convergent evolution of the global sponge microbiome. Nat Commun.

[56] Lima-Mendez G, Van Helden J, Toussaint A, Leplae R (2008). Reticulate representation of evolutionary and functional relationships between phage genomes. Mol Biol Evol.

[57] Bavestrello G, Burlando B, Sara M (1988). The architecture of the canal systems of *Petrosia ficiformis* and *Chondrosia reniformis* studied by corrosion casts (*Porifera*, *Demospongiae*). Zoomorphology.

[58] Van Soest RWM, Boury-Esnault N, Hooper JNA, Rützler K, de Voogd NJ, Alvarez B et al. World porifera database. 2019.

[59] Oh JH, Alexander LM, Pan M, Schueler KL, Keller MP, Attie AD (2019). Dietary fructose and microbiota-derived short-chain fatty acids promote bacteriophage production in the gut symbiont Lactobacillus reuteri. Cell Host Microbe.

[60] De Paepe M, Tournier L, Moncaut E, Son O, Langella P, Petit MA (2016). Carriage of lambda latent virus is costly for its bacterial host due to frequent reactivation in monoxenic mouse intestine. PLoS Genet.

[61] Barr JJ, Auro R, Furlan M, Whiteson KL, Erb ML, Pogliano J (2013). Bacteriophage adhering to mucus provide a non-host-derived immunity. PNAS.

[62] Roux S (2019). A viral ecogenomics framework to uncover the secrets of nature’s “microbe whisperers”. mSystems.

[63] Paez-Espino D, Roux S, Chen IA, Palaniappan K, Ratner A, Chu K (2019). IMG/VR v.2.0: an integrated data management and analysis system for cultivated and environmental viral genomes. Nucleic Acids Res.

[64] Deng L, Ignacio-Espinoza JC, Gregory AC, Poulos BT, Weitz JS, Hugenholtz P (2014). Viral tagging reveals discrete populations in *Synechococcus* viral genome sequence space. Nature.

[65] Džunková M, Low SJ, Daly JN, Deng L, Rinke C, Hugenholtz P (2019). Defining the human gut host–phage network through single-cell viral tagging.. Nat Microbiol..

[66] Marbouty M, Baudry L, Cournac A, Koszul R (2017). Scaffolding bacterial genomes and probing host-virus interactions in gut microbiome by proximity ligation (chromosome capture) assay. Sci Adv.

[67] Sullivan MB, Waterbury JB, Chisholm SW (2003). Cyanophages infecting the oceanic cyanobacterium Prochlorococcus. Nature.

[68] de Jonge PA, Nobrega FL, Brouns SJJ, Dutilh BE (2019). Molecular and evolutionary determinants of bacteriophage host range. Trends Microbiol.

[69] Kauffman KM, Hussain FA, Yang J, Arevalo P, Brown JM, Chang WK (2018). A major lineage of non-tailed dsDNA viruses as unrecognized killers of marine bacteria.. Nature.

[70] Flores CO, Valverde S, Weitz JS (2013). Multi-scale structure and geographic drivers of cross-infection within marine bacteria and phages. ISME J.

[71] Soffer N, Zaneveld J, Vega Thurber R (2015). Phage-bacteria network analysis and its implication for the understanding of coral disease. Environ Microbiol.

[72] Tzipilevich E, Habusha M, Ben-Yehuda S (2017). Acquisition of phage sensitivity by bacteria through exchange of phage receptors. Cell.

[73] Battich N, Stoeger T, Pelkmans L (2013). Image-based transcriptomics in thousands of single human cells at single-molecule resolution. Nat Methods.

[74] Li X-Y, Lachnit T, Fraune S, Bosch TCG, Traulsen A, Sieber M (2017). Temperate phages as self-replicating weapons in bacterial competition. J R Soc Interface.

[75] Pascelli C, Laffy PW, Kupresanin M, Ravasi T, Webster NS (2018). Morphological characterization of virus-like particles in coral reef sponges. PeerJ.

[76] Sime-Ngando T (2014). Environmental bacteriophages: viruses of microbes in aquatic ecosystems. Front Microbiol.

[77] Knowles B, Silveira CB, Bailey BA, Barott K, Cantu VA, Cobián-Güemes AG (2016). Lytic to temperate switching of viral communities. Nature.

[78] Duerkop BA, Clements CV, Rollins D, Rodrigues JLM, Hooper LV. A composite bacteriophage alters colonization by an intestinal commensal bacterium. PNAS. 2012;109:17621–6. 10.1073/pnas.1206136109 PMC3491505 23045666

[79] Thingstad TF, Vage S, Storesund JE, Sandaa RA, Giske J (2014). A theoretical analysis of how strain-specific viruses can control microbial species diversity. PNAS.

[80] Morella NM, Gomez AL, Wang G, Leung MS, Koskella B (2018). The impact of bacteriophages on phyllosphere bacterial abundance and composition. Mol Ecol.

[81] Wattam AR, Davis JJ, Assaf R, Boisvert S, Brettin T, Bun C (2017). Improvements to PATRIC, the all-bacterial Bioinformatics Database and Analysis Resource Center. Nucleic Acids Res.

