# Supplementary material for: Lifestyle of sponge symbiont phages by host prediction and correlative microscopy
Source: ISME J. 2021 Feb 18;15(7):2001–11. doi: 10.1038/s41396-021-00900-6 (PMC8245591; doi:10.1038/s41396-021-00900-6)
Supplement: Supplementary file 1 — Supplementary Information [file 41396_2021_900_MOESM1_ESM.pdf]

Lifestyle of sponge symbiont phages by host prediction and correlative microscopy

Jahn M.T. [1,2,&], Lachnit T.[3], Markert S.M.[4], Stigloher C.[4], Pita L.[1], Ribes M.[5], Dutilh B.E.[6] & Hentschel U.[1,3]

[1] GEOMAR Helmholtz Centre for Ocean Research Kiel, Kiel, Germany
[2] Current address: Department of Zoology and Department of Biochemistry, University of Oxford, Oxford, UK
[3] Christian-Albrechts-University of Kiel, Kiel, Germany
[4] Imaging Core Facility, Biocenter, University of Würzburg, Würzburg, Germany
[5] Institut de Ciències del Mar (ICM-CSIC), Barcelona, Spain
[6] Theoretical Biology and Bioinformatics, Utrecht University, Utrecht, The Netherlands
[&] Correspondence: martin.jahn@zoo.ox.ac.uk

Supplementary Information

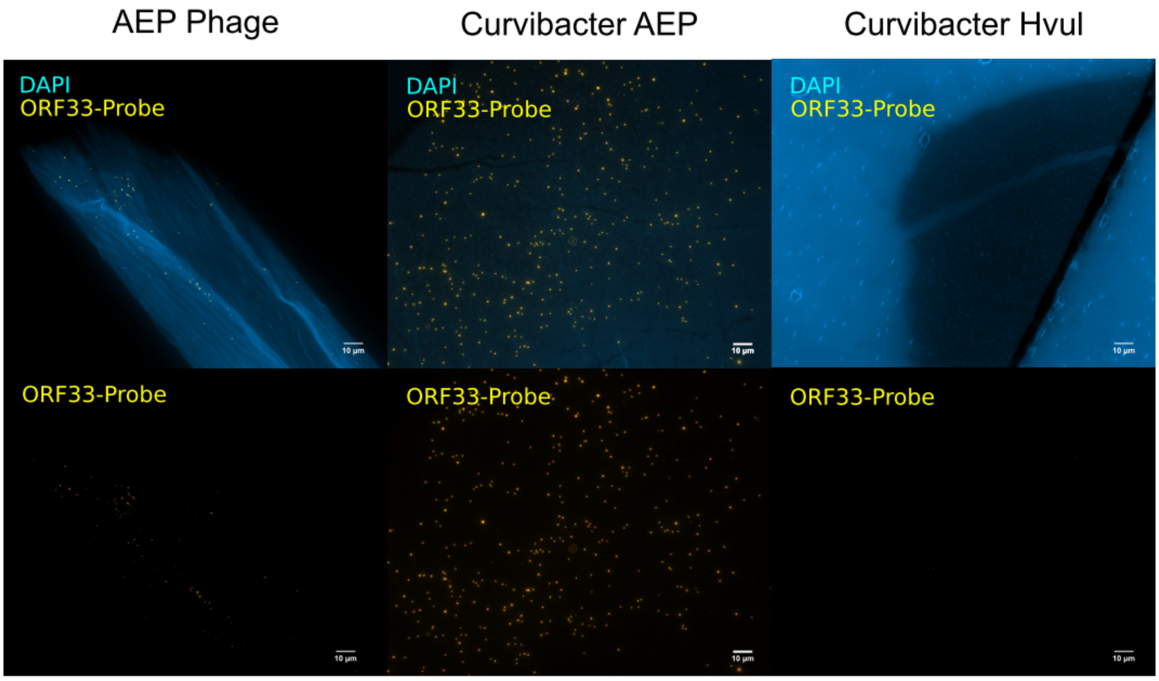

Figure S1: Branched-DNA fluorescence *in situ* hybridization system testing. Multiple probes (Cy3, yellow, see methods) targeting Curvibacter AEP ORF33 encoding the major capsid protein (VOG1887) were applied to purified Curvibacter AEP virions attached to a fibre matrix (blue autofluorescence), Curvibacter AEP (lysogens), Curvibacter Hvul (non lysogen) sharing 95.4% ANI.

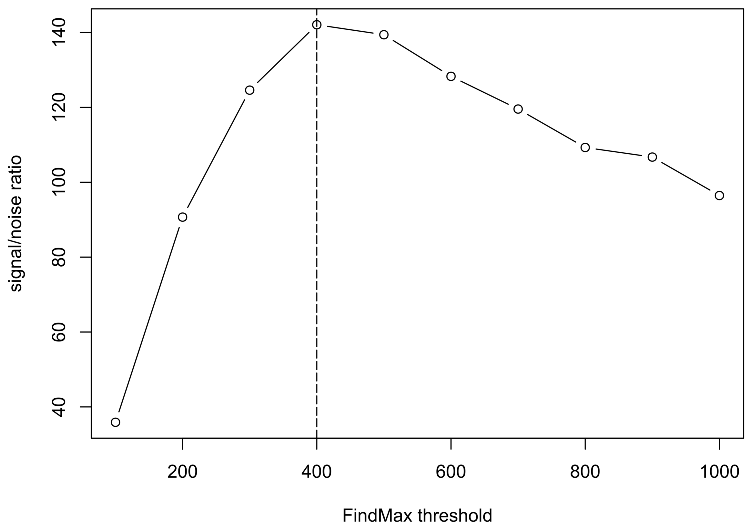

Figure S2: Optimizing "signal-to-noise" ratios for automatic signal detection. Impact of successive changes of ImageJ-FindMax threshold on positive:negative control ratio. A threshold of 400 units allowed a maximal signal, as indicated by the dashed line.

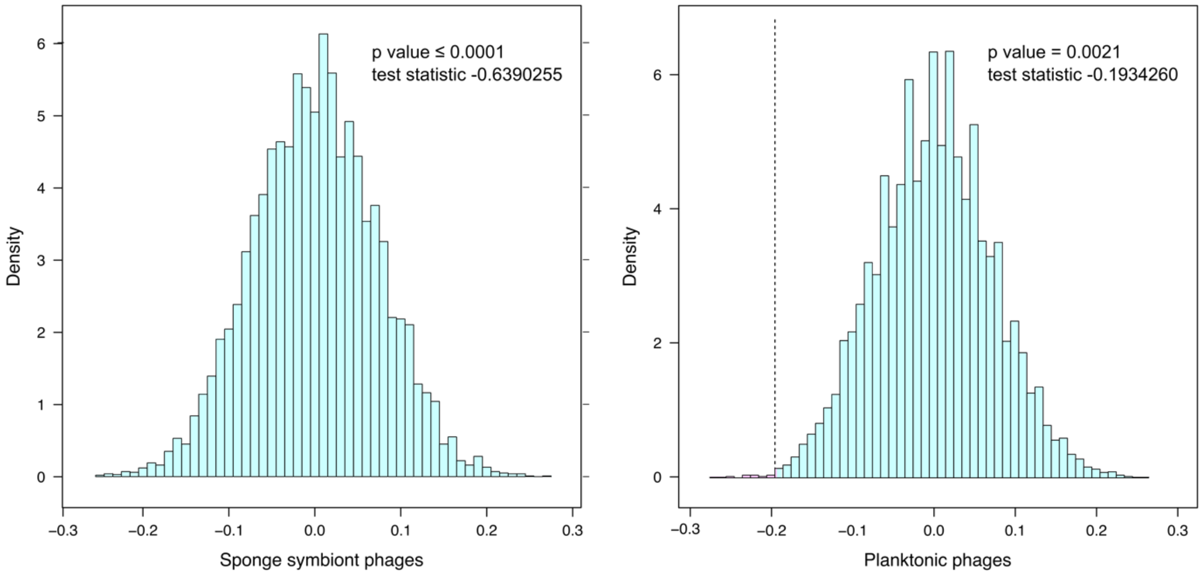

Figure S3. Comparison of the test statistics for symbiont phages and planktophages against randomised networks. To infer significant stratification of phage-bacteria pairings between sponge associated and the planktonic environment the complete network was compared to N=10,000 networks where labels were randomised using a custom script. Related to Figure 1.

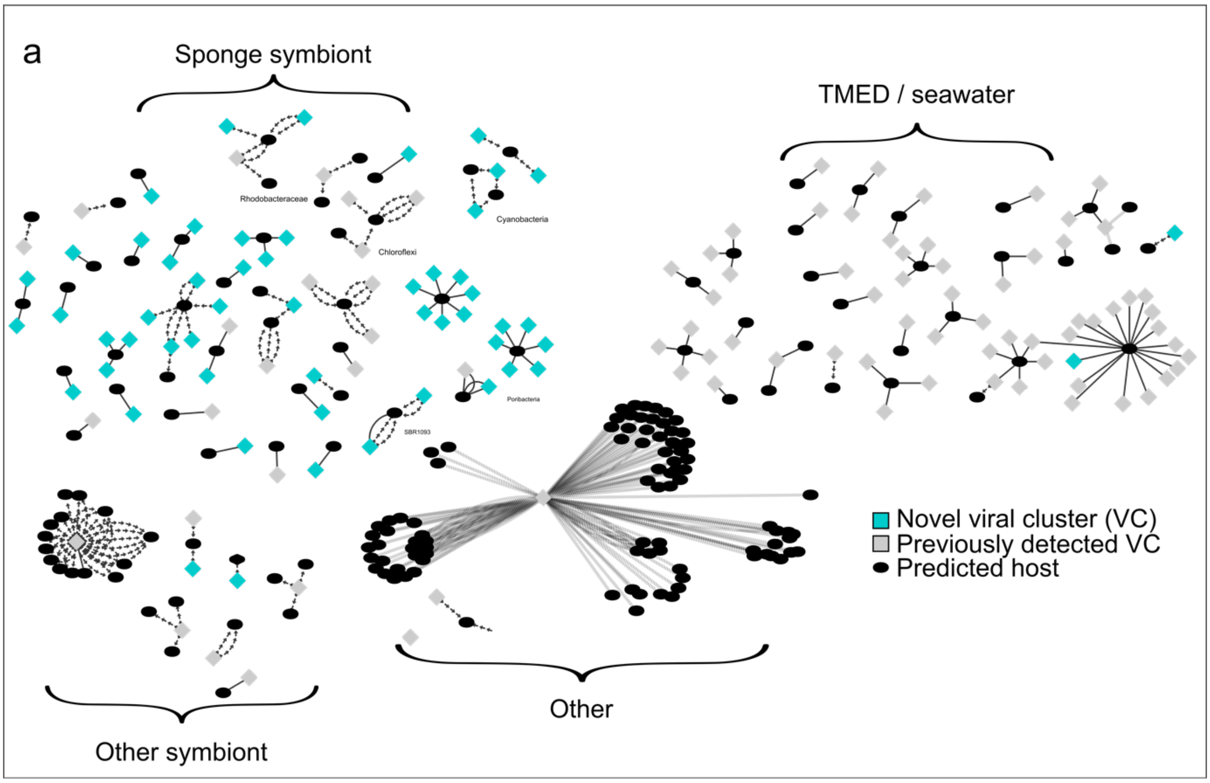

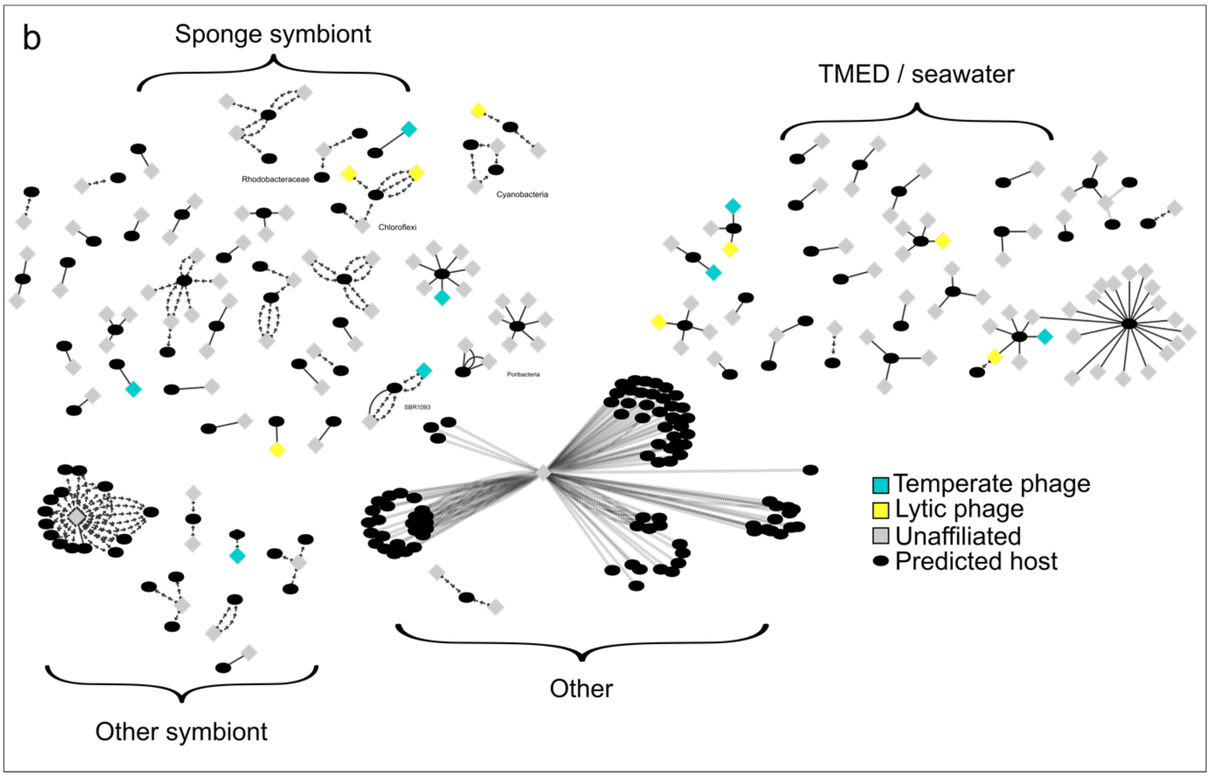

Figure S4: Full phage-bacteria infection network providing additional information on phage (a) taxonomic novelty and (b) predicted replication strategy.

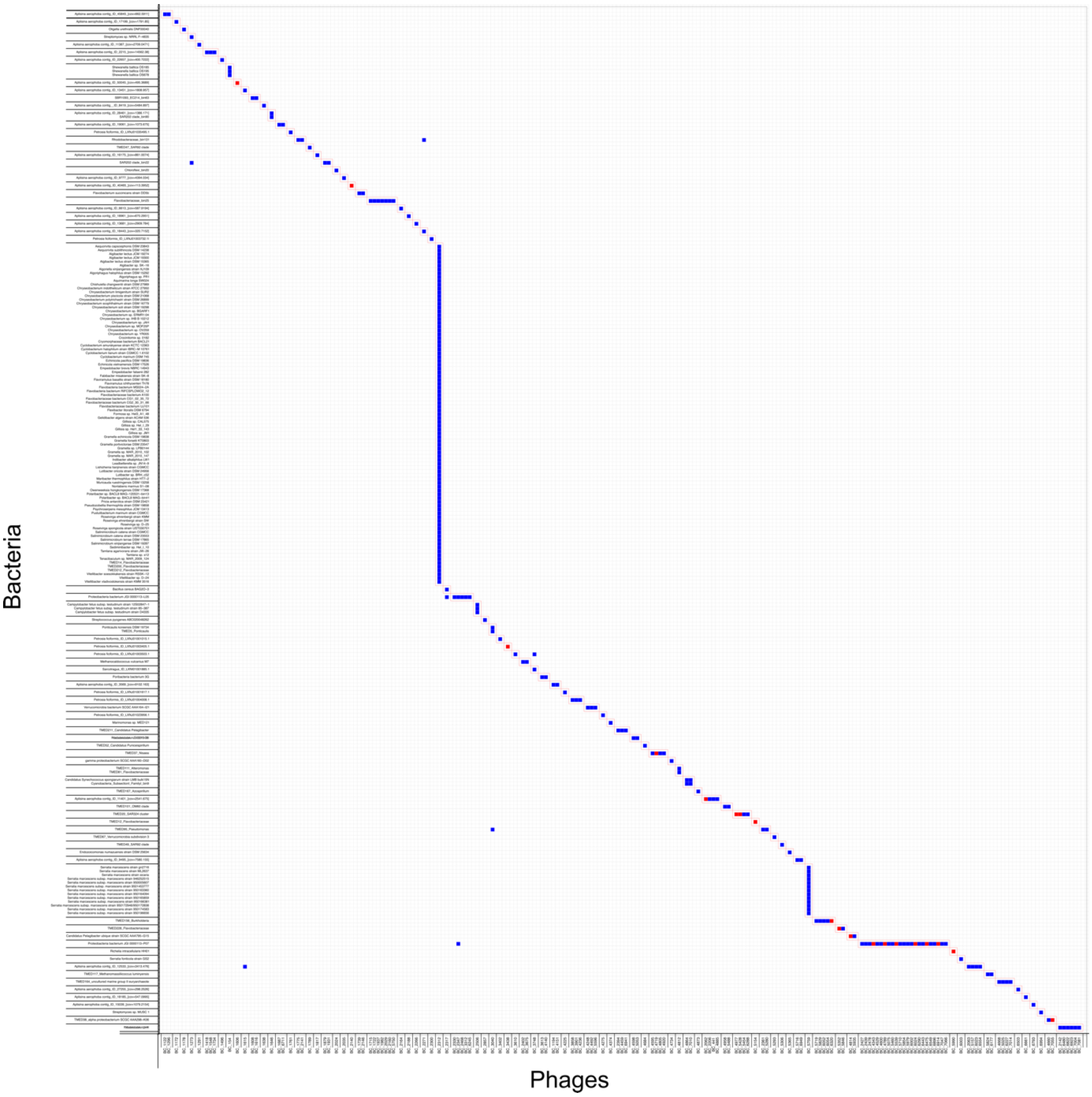

Figure S5. Complete Host–phage interaction matrix of sponge associated microbiota. Blue cells denote *in silico* host predictions based on CRISPR-spacer-, homology-, and tRNA matches against a custom database of sponge microbial sequences, 97,941 PATRIC genomes (Wattam et al 2017) and 290 Tara Oceans bins from bacterioplankton of the Mediterranean Sea (TMED; Tully et al (2017)). Blue boxes are predicted interactions that meet all set quality criteria, red interactions were excluded from the final set representing homology matches where phage genomes aligned more than 50% relative to the bacterial target genome contig.

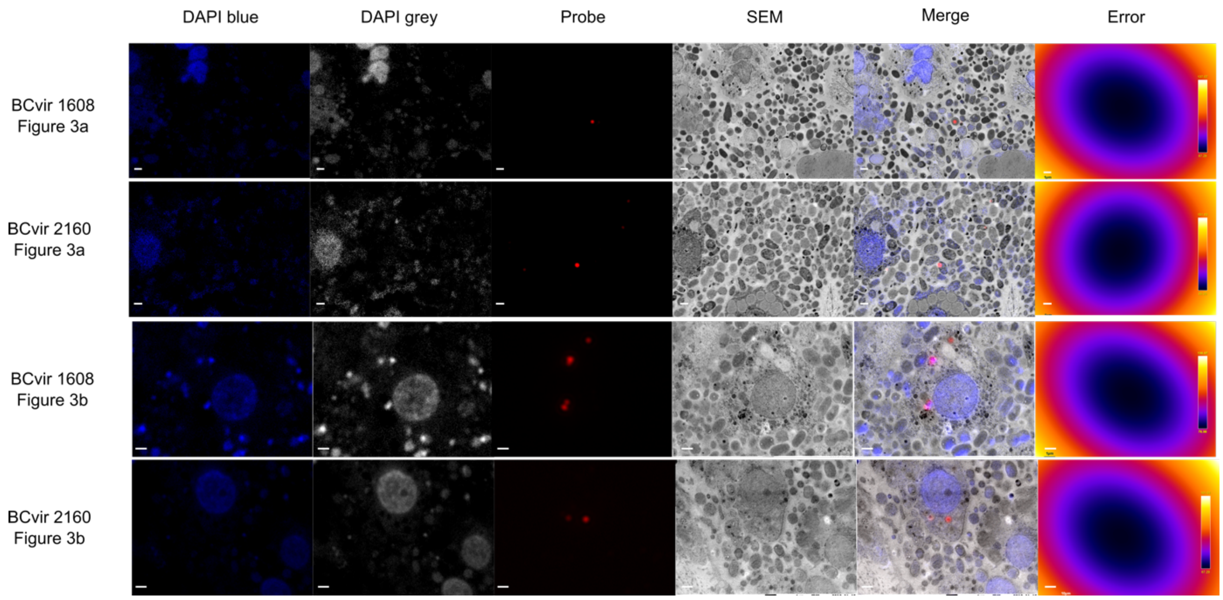

Figure S6 Separate channels and predicted error map of set correlation for Fig. 3. Error indicates the registration error maps generated by eC-CLEM and shows the distribution of predicted alignment error for rigid set correlation (fluorescence versus scanning electron microscopy). Colour scale indicates the predicted deviation in nm. Scale bars 1 µm.

Table S1. Sampling metadata.

species	used for	location	date	time	temperature	depth	lat	long
A. aerophoba	microscopy	cala d'Aigüafreda	4. October 2016	noon	21°C	3m	41°57'51.3"N	3°13'39.8"E
A. aerophoba	virome	Port Lligat	12. July 2016	noon	23.3°C	3m	42°17'50.8"N	3°17'20.4"E
P. ficiformis	virome	Reserva Natural de Montgrí, Illes Medes i Baix Ter	18. July 2016	noon	21°C	15m	42°04'56.97"N	3°12'00.09"E
C. reniformis	virome	Reserva Natural de Montgrí, Illes Medes i Baix Ter	18. July 2016	noon	21°C	15m	42°04'56.97"N	3°12'00.09"E
A. oroides	virome	Reserva Natural de Montgrí, Illes Medes i Baix Ter	18. July 2016	noon	21°C	15m	42°04'56.97"N	3°12'00.09"E

Table S3. Kruskal–Wallis test followed by Dunn's Post hoc-Tests with Benjamini-Hochberg false discovery rate correction.

pairwise comparisons	Z statistic (adjusted p value)
BC_1608 - BC_2142	0.141592 (0.4437)
BC_1608 - BC_2160	-0.161975 (0.4841)
BC_2142 - BC_2160	-0.265500 (0.4941)
BC_1608 - neg_control	3.665383 (0.0002)*
BC_2142 - neg_control	3.324442 (0.0006)*
BC_2160 - neg_control	3.350797 (0.0007)*
BC_1608 - pos_control	-7.922506 (0.0000)*
BC_2142 - pos_control	-7.421882 (0.0000)*
BC_2160 - pos_control	-6.666282 (0.0000)*
neg_control - pos_control	-9.349236 (0.0000)*
alpha = 0.05	
Reject Ho if p <= alpha/2	

Table S4. Kruskal–Wallis tests, pairwise tissue comparison.

pairwise comparisons	chi-squared	df	p-value
BC_1608: mesohyl - pinacoderm	0.24758	1	0.6188
BC_2142: mesohyl - pinacoderm	0.58098	1	0.4459
BC_2160: mesohyl - pinacoderm	1.1738	1	0.2786
